# μ-Carbonato-κ^4^ *O*,*O*′:*O*′,*O*′′-bis­{[2′-(di-*tert*-butyl­phosphan­yl)biphenyl-2-yl-κ^2^ *P*,*C* ^1^]palladium(II)} dichloro­methane monosolvate

**DOI:** 10.1107/S1600536812048702

**Published:** 2012-11-30

**Authors:** Alfred Muller, Cedric W. Holzapfel

**Affiliations:** a Department of Chemistry, University of Johannesburg (APK Campus), PO Box 524, Auckland Park, Johannesburg, 2006, South Africa

## Abstract

The title compound, [(μ_2_-CO_3_){Pd(P(*t*-C_4_H_9_)_2_(C_12_H_8_)}_2_]·CH_2_Cl_2_, the first CO_3_-bridged palladium dimer complex reported to date, was obtained while preparing the Pd^0^ complex with (2-biphen­yl)P(^*t*^Bu)_2_. In the crystal, each palladium dimer is accompanied by a dichloro­methane solvent mol­ecule. Coordination of the carbonate and chelated phosphane ligands gives distorted square-planar environments at the Pd atoms. Important geometrical parameters include Pd—P(av.) = 2.2135 (4) Å, Pd—C(av.) = 1.9648 (16) Å and P—Pd—C = 84.05 (5) and 87.98 (5)°, and O—Pd—O′ = 60.56 (4) and 61.13 (4)°. Bonding with the carbonate O atoms shows values of 2.1616 (11) and 2.1452 (11) Å for the Pd—O—Pd bridge, whereas other Pd—O distances are slightly longer at 2.2136 (11) and 2.1946 (11) Å. One of the *tert*-butyl groups is disordered over two set of sites with an occupancy ratio of 0.723 (6):0.277 (6). Weak C—H⋯O interactions are observed propagating the molecules along the [100] direction.

## Related literature

For catalytic studies on palladium complexes [Pd_2_(dba)_3_] or [Pd(dba)_3_)], where dba = dibenzyl­ideneacetone, in combination with 2-biphenyl-di-*tert*-butyl­phosphane, see: Barlenga *et al.* (2007 ▶); Christman *et al.* (2006 ▶); Ohmura *et al.* (2008 ▶); Williams *et al.* (2008 ▶); Omondi *et al.* (2011 ▶); de Pater *et al.* (2005 ▶).
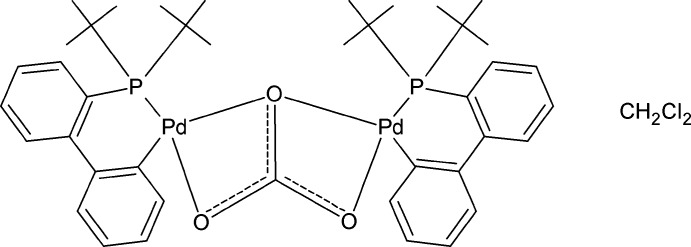


## Experimental

### 

#### Crystal data

[Pd_2_(CO_3_)(C_20_H_26_P)_2_]·CH_2_Cl_2_
*M* *_r_* = 952.49
Triclinic, 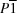

*a* = 7.5459 (6) Å
*b* = 14.6579 (11) Å
*c* = 18.7078 (14) Å
α = 83.866 (2)°
β = 86.457 (2)°
γ = 86.478 (2)°
*V* = 2050.3 (3) Å^3^
*Z* = 2
Mo *K*α radiation
μ = 1.12 mm^−1^
*T* = 100 K
0.42 × 0.1 × 0.06 mm

#### Data collection

Bruker APEX DUO 4K CCD diffractometer
Absorption correction: multi-scan (*SADABS*; Bruker, 2008 ▶) *T*_min_ = 0.650, *T*_max_ = 0.936
86748 measured reflections
10165 independent reflections
8987 reflections with *I* > 2σ(*I*)
*R*_int_ = 0.033

#### Refinement

*R*[*F* ^2^ > 2σ(*F* ^2^)] = 0.020
*wR*(*F* ^2^) = 0.044
*S* = 1.03
10165 reflections
503 parameters
78 restraints
H-atom parameters constrained
Δρ_max_ = 0.56 e Å^−3^
Δρ_min_ = −0.48 e Å^−3^

### 

Data collection: *APEX2* (Bruker, 2011 ▶); cell refinement: *SAINT* (Bruker, 2008 ▶); data reduction: *SAINT* and *XPREP* (Bruker, 2008 ▶); program(s) used to solve structure: *SIR97* (Altomare *et al.*, 1999 ▶); program(s) used to refine structure: *SHELXL97* (Sheldrick, 2008 ▶); molecular graphics: *DIAMOND* (Brandenburg & Putz, 2005 ▶); software used to prepare material for publication: *publCIF* (Westrip, 2010 ▶) and *WinGX* (Farrugia, 2012 ▶).

## Supplementary Material

Crystal structure: contains datablock(s) global, I. DOI: 10.1107/S1600536812048702/yk2080sup1.cif

Structure factors: contains datablock(s) I. DOI: 10.1107/S1600536812048702/yk2080Isup2.hkl

Additional supplementary materials:  crystallographic information; 3D view; checkCIF report

## Figures and Tables

**Table 1:** Hydrogen-bond geometry (Å, °)

*D*—H⋯*A*	*D*—H	H⋯*A*	*D*⋯*A*	*D*—H⋯*A*
C18—H18*A*⋯O1	0.98	2.53	3.362 (2)	143
C20—H20*C*⋯O1	0.98	2.27	3.182 (2)	154
C22—H22⋯O2	0.95	2.43	3.030 (2)	121
C35—H35*A*⋯O2^i^	0.98	2.43	3.358 (2)	159
C38—H38*C*⋯O3	0.98	2.37	3.223 (2)	146
C40—H40*A*⋯O3	0.98	2.51	3.377 (2)	147
C42—H42*A*⋯O3^ii^	0.99	2.40	3.190 (2)	136

Symmetry codes: (i) 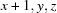; (ii) 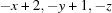.

**Fig. 1.**
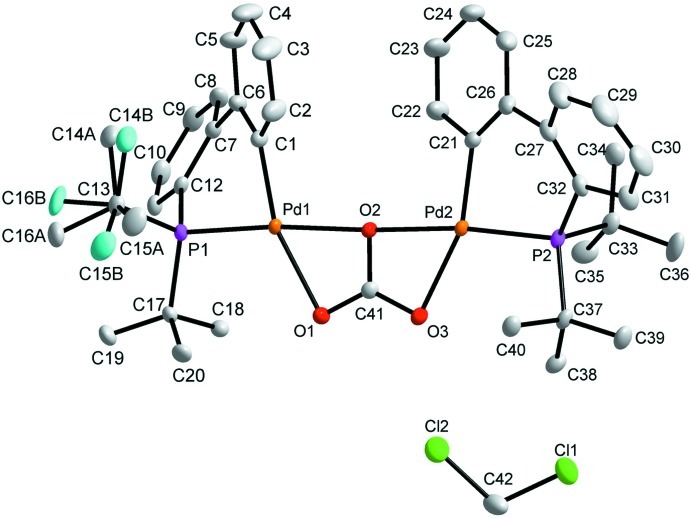
View of title compound showing displacement ellipsoids drawn at a 30% probability level and atom-numbering scheme. Secondary component of disordered atoms indicated in cyan. Hydrogen atoms have been omitted for clarity.

## supplementary crystallographic information

### Comment

Zero-valent palladium complexes play a central role as catalysts in organic synthesis. The complexes Pd_2_(dba)_3_ or Pd(dba)_3_, where dba = dibenzylideneacetone, in combination with 2-biphenyl-di-*tert*-butylphosphane constitutes particularly powerful catalytic systems (Barlenga *et al.*, 2007; Christman *et al.*, 2006; Ohmura *et al.*, 2008). These catalysts were also successfully applied in the hydromethoxycarbonylation of alkenes under conditions previously described (Williams *et al.*, 2008; Omondi *et al.*, 2011). However, the application of these catalysts in the hydromethoxycarbonylation of styrene suggested that dibenzylideneacetone (dba), like other electron deficient alkenes, may have an inhibitory effect, possibly due to its strong co-ordination to Pd^0^ (de Pater *et al.*, 2005). We therefore removed dba from our catalyst preparation. This resulted in substantial enhancement of reaction rates. The putative catalyst, a complex of Pd^0^ with (2-biphenyl)P(*^t^*Bu)_2_, could not be crystallized. Exposure of the solution to the air resulted in crystals of the title compound, the structure of which was established by single-crystal X-ray diffraction. The formation of the title compound can be rationalized in terms of reaction of the putative complex with oxygen and carbon dioxide.

Molecules of the title compound (Figure 1, Scheme 1) occupy general positions in the *P*1 space group. The coordination environment for each Pd center is distorted square-planar with P—Pd—C = 84.05/87.98 (5)°, O—Pd—O' = 60.56/61.13 (4)° due to the strained four membered chelation of the carbonato ligand to each metal center. Bonding to the carbonato oxygen atoms shows values of 2.1616 (11)/2.1452 (11) Å for Pd—O—Pd, whereas the Pd—O distances are slightly longer at 2.2136 (11)/2.1946 (11) Å. Weak C—H···O interactions are observed and the resulting packing causes a disorder of one the tertiary butyl groups which was treated appropriately (see the Refinement section for details).

### Experimental

To a stirred solution of Pd(dba)_2_ (574 mg, 1 mmol) in CH_2_Cl_2_ (25 ml) at room temperature under argon was added (2-biphenyl)P(*^t^*Bu)_2_ (596 mg, 2 mmol). Stirring was continued for 24 hr. During this period the color of the solution changed from dark red to a light orange. The solvent was removed *in vacuo* and the residue chromatographed over silica (Davisil, 25 g) using gradient elution (0–5% acetone in CH_2_Cl_2_). After all of the dba was eluted from the column (150 ml), as indicated by TLC, the remaining absorbed material was eluted using 20% acetone in CH_2_Cl_2_ (75 ml). Evaporation of the solvent *in vacuo* left a colorless amorphous residue (640 mg) which showed a major ^31^P-NMR signal at -13.5 p.p.m. A solution of the residue in 5:1 cyclohexane-CH_2_Cl_2_ was exposed to the atmosphere. As the solvent evaporated, crystals of the title compound were deposited. The colorless crystals (443 mg) were collected after 24 hrs. Good quality crystals (mp. 144–147°C, decomp.) were obtained by exposing a solution of the title compound in CH_2_Cl_2_ to vapors of ether.

### Refinement

All methyl and aromatic hydrogen atoms were positioned in geometrically idealized positions with C—H = 0.96 Å and 0.93 Å, respectively. Aromatic hydrogen atoms were allowed to ride on their parent atoms with *U*_iso_(H) = 1.2*U*_eq_, and for methyl hydrogen atoms *U*_iso_(H) = 1.5*U*_eq_ was used. The initial positions of methyl hydrogen atoms were located from a Fourier difference map and refined as fixed rotor. Discrepant reflection [001] was removed at the final stages of refinement. A tertiary butyl on P1 showed large thermal ellipsoids and was refined with the methyl groups disordered over two positions. The 1,2 and 1,3 distance restraints (SADI), as well as ellipsoid displacement restraints (SIMU and DELU), were applied to keep refinement stable and reasonable. Occupation parameters of the disordered atoms refined to a 0.72245:0.27755 ratio for parts A and B, respectively. All the above restraints were applied with the default standard deviations. The highest residual peak of 0.56 Å^-3^ is 0.85 Å from C4 and the deepest hole of -0.48 Å^-3^ 0.81 Å from Pd1, both representing no physical meaning.

### Crystal data

**Table:**

[Pd_2_(CO_3_)(C_20_H_26_P)_2_]·CH_2_Cl_2_	*Z* = 2
*M**_r_* = 952.49	*F*(000) = 972
Triclinic, *P*1	*D*_x_ = 1.543 Mg m^−^^3^
Hall symbol: -P 1	Mo *K*α radiation, λ = 0.71073 Å
*a* = 7.5459 (6) Å	Cell parameters from 9940 reflections
*b* = 14.6579 (11) Å	θ = 2.5–28.2°
*c* = 18.7078 (14) Å	µ = 1.12 mm^−^^1^
α = 83.866 (2)°	*T* = 100 K
β = 86.457 (2)°	Needle, yellow
γ = 86.478 (2)°	0.42 × 0.1 × 0.06 mm
*V* = 2050.3 (3) Å^3^	

### Data collection

**Table:**

Bruker APEX DUO 4K CCD diffractometer	10165 independent reflections
Graphite monochromator	8987 reflections with *I* > 2σ(*I*)
Detector resolution: 8.4 pixels mm^-1^	*R*_int_ = 0.033
φ and ω scans	θ_max_ = 28.3°, θ_min_ = 1.7°
Absorption correction: multi-scan (*SADABS*; Bruker, 2008)	*h* = −10→9
*T*_min_ = 0.650, *T*_max_ = 0.936	*k* = −19→19
86748 measured reflections	*l* = −24→24

### Refinement

**Table:**

Refinement on *F*^2^	Primary atom site location: structure-invariant direct methods
Least-squares matrix: full	Secondary atom site location: difference Fourier map
*R*[*F*^2^ > 2σ(*F*^2^)] = 0.020	Hydrogen site location: inferred from neighbouring sites
*wR*(*F*^2^) = 0.044	H-atom parameters constrained
*S* = 1.03	*w* = 1/[σ^2^(*F*_o_^2^) + (0.0104*P*)^2^ + 2.0663*P*] where *P* = (*F*_o_^2^ + 2*F*_c_^2^)/3
10165 reflections	(Δ/σ)_max_ = 0.004
503 parameters	Δρ_max_ = 0.56 e Å^−^^3^
78 restraints	Δρ_min_ = −0.48 e Å^−^^3^

### Special details

**Table:**

Experimental. The intensity data was collected on a Bruker Apex DUO 4 K CCD diffractometer using an exposure time of 10 s/frame. A total of 4968 frames were collected with a frame width of 0.5° covering up to θ = 28.31° with 99.8% completeness accomplished.
Geometry. All e.s.d.'s (except the e.s.d. in the dihedral angle between two l.s. planes) are estimated using the full covariance matrix. The cell e.s.d.'s are taken into account individually in the estimation of e.s.d.'s in distances, angles and torsion angles; correlations between e.s.d.'s in cell parameters are only used when they are defined by crystal symmetry. An approximate (isotropic) treatment of cell e.s.d.'s is used for estimating e.s.d.'s involving l.s. planes.
Refinement. Refinement of *F*^2^ against ALL reflections. The weighted *R*-factor *wR* and goodness of fit *S* are based on *F*^2^, conventional *R*-factors *R* are based on *F*, with *F* set to zero for negative *F*^2^. The threshold expression of *F*^2^ > σ(*F*^2^) is used only for calculating *R*-factors(gt) *etc*. and is not relevant to the choice of reflections for refinement. *R*-factors based on *F*^2^ are statistically about twice as large as those based on *F*, and *R*- factors based on ALL data will be even larger.

### Fractional atomic coordinates and isotropic or equivalent isotropic displacement parameters (Å^2^)

**Table:**

	*x*	*y*	*z*	*U*_iso_*/*U*_eq_	Occ. (<1)
Pd1	0.883397 (16)	0.921980 (7)	0.215760 (6)	0.01235 (3)	
Pd2	1.238700 (16)	0.682903 (8)	0.254095 (6)	0.01277 (3)	
P1	0.71344 (5)	1.04810 (3)	0.18774 (2)	0.01269 (8)	
P2	1.43043 (6)	0.56268 (3)	0.26182 (2)	0.01438 (8)	
C1	0.8211 (2)	0.94764 (11)	0.31550 (9)	0.0188 (3)	
C2	0.7610 (3)	0.87228 (13)	0.36043 (10)	0.0304 (4)	
H2	0.7504	0.8157	0.341	0.037*	
C3	0.7167 (4)	0.87820 (14)	0.43276 (11)	0.0423 (6)	
H3	0.672	0.8268	0.4621	0.051*	
C4	0.7378 (4)	0.95913 (15)	0.46222 (11)	0.0454 (6)	
H4	0.7102	0.9633	0.512	0.054*	
C5	0.7994 (3)	1.03403 (14)	0.41859 (10)	0.0343 (5)	
H5	0.816	1.0891	0.4392	0.041*	
C6	0.8381 (2)	1.03058 (11)	0.34455 (9)	0.0200 (3)	
C7	0.8777 (2)	1.11743 (11)	0.29964 (9)	0.0173 (3)	
C8	0.9715 (2)	1.18333 (12)	0.32779 (10)	0.0238 (4)	
H8	1.016	1.1699	0.3744	0.029*	
C9	1.0016 (3)	1.26786 (12)	0.28960 (11)	0.0269 (4)	
H9	1.0669	1.3113	0.3097	0.032*	
C10	0.9359 (2)	1.28830 (11)	0.22230 (10)	0.0237 (4)	
H10	0.9548	1.3463	0.1959	0.028*	
C11	0.8421 (2)	1.22408 (11)	0.19310 (9)	0.0179 (3)	
H11	0.7965	1.2391	0.1469	0.022*	
C12	0.8134 (2)	1.13801 (10)	0.23007 (8)	0.0147 (3)	
C13	0.4695 (2)	1.04852 (11)	0.21867 (9)	0.0194 (3)	
C14A	0.4407 (4)	1.0692 (3)	0.29654 (16)	0.0264 (7)	0.723 (6)
H14A	0.4856	1.1293	0.3016	0.04*	0.723 (6)
H14B	0.5045	1.0217	0.3276	0.04*	0.723 (6)
H14C	0.3134	1.0698	0.3106	0.04*	0.723 (6)
C15A	0.4089 (4)	0.9496 (2)	0.21425 (19)	0.0311 (8)	0.723 (6)
H15A	0.2824	0.947	0.2292	0.047*	0.723 (6)
H15B	0.4784	0.9057	0.2461	0.047*	0.723 (6)
H15C	0.4278	0.934	0.1646	0.047*	0.723 (6)
C16A	0.3543 (4)	1.1164 (2)	0.17218 (16)	0.0284 (8)	0.723 (6)
H16A	0.3564	1.0975	0.1234	0.043*	0.723 (6)
H16B	0.4004	1.1778	0.1701	0.043*	0.723 (6)
H16C	0.2319	1.1179	0.193	0.043*	0.723 (6)
C14B	0.4398 (12)	1.0218 (7)	0.3003 (4)	0.032 (2)	0.277 (6)
H14D	0.5083	1.0607	0.3265	0.047*	0.277 (6)
H14E	0.4791	0.9572	0.312	0.047*	0.277 (6)
H14F	0.3131	1.0305	0.3144	0.047*	0.277 (6)
C15B	0.3614 (10)	0.9902 (6)	0.1786 (5)	0.033 (2)	0.277 (6)
H15D	0.2446	0.983	0.2038	0.05*	0.277 (6)
H15E	0.4229	0.9297	0.1761	0.05*	0.277 (6)
H15F	0.3461	1.02	0.1298	0.05*	0.277 (6)
C16B	0.3913 (10)	1.1520 (4)	0.2068 (5)	0.031 (2)	0.277 (6)
H16D	0.4065	1.1747	0.1557	0.046*	0.277 (6)
H16E	0.4548	1.1902	0.2354	0.046*	0.277 (6)
H16F	0.2646	1.1547	0.2218	0.046*	0.277 (6)
C17	0.7439 (2)	1.07393 (11)	0.08665 (8)	0.0167 (3)	
C18	0.9443 (2)	1.08395 (11)	0.06970 (8)	0.0182 (3)	
H18A	1.0108	1.0301	0.0923	0.027*	
H18B	0.9803	1.1395	0.0885	0.027*	
H18C	0.9695	1.0887	0.0175	0.027*	
C19	0.6405 (3)	1.15768 (13)	0.04808 (10)	0.0272 (4)	
H19A	0.6463	1.211	0.0751	0.041*	
H19B	0.516	1.1434	0.0454	0.041*	
H19C	0.6937	1.1716	−0.0007	0.041*	
C20	0.6905 (2)	0.98841 (12)	0.05387 (9)	0.0227 (4)	
H20A	0.7169	0.9959	0.0017	0.034*	
H20B	0.5629	0.9809	0.0639	0.034*	
H20C	0.7577	0.9339	0.0752	0.034*	
C21	1.2346 (2)	0.67858 (12)	0.35946 (9)	0.0191 (3)	
C22	1.2133 (3)	0.76783 (13)	0.37983 (10)	0.0311 (4)	
H22	1.2043	0.8183	0.3437	0.037*	
C23	1.2050 (4)	0.78418 (16)	0.45139 (11)	0.0432 (6)	
H23	1.1889	0.8453	0.4641	0.052*	
C24	1.2202 (3)	0.71151 (17)	0.50420 (11)	0.0419 (6)	
H24	1.2177	0.7223	0.5534	0.05*	
C25	1.2389 (3)	0.62345 (15)	0.48512 (10)	0.0335 (5)	
H25	1.249	0.5737	0.5219	0.04*	
C26	1.2437 (2)	0.60435 (12)	0.41274 (9)	0.0218 (4)	
C27	1.2734 (2)	0.50673 (12)	0.39809 (9)	0.0220 (4)	
C28	1.2097 (3)	0.43839 (14)	0.44993 (11)	0.0338 (5)	
H28	1.1323	0.456	0.4887	0.041*	
C29	1.2561 (3)	0.34662 (15)	0.44627 (12)	0.0395 (5)	
H29	1.2071	0.3017	0.4811	0.047*	
C30	1.3731 (3)	0.32008 (13)	0.39228 (12)	0.0345 (5)	
H30	1.4139	0.2575	0.3919	0.041*	
C31	1.4315 (3)	0.38559 (12)	0.33809 (10)	0.0259 (4)	
H31	1.511	0.3669	0.3003	0.031*	
C32	1.3757 (2)	0.47853 (11)	0.33788 (9)	0.0187 (3)	
C33	1.6607 (2)	0.59679 (11)	0.27585 (9)	0.0201 (3)	
C34	1.6696 (2)	0.61526 (12)	0.35490 (10)	0.0237 (4)	
H34A	1.6424	0.5595	0.3864	0.036*	
H34B	1.5828	0.6652	0.3655	0.036*	
H34C	1.7893	0.6328	0.3631	0.036*	
C35	1.6989 (3)	0.68592 (13)	0.22825 (10)	0.0273 (4)	
H35A	1.8134	0.7073	0.2396	0.041*	
H35B	1.6045	0.7329	0.2373	0.041*	
H35C	1.7031	0.6745	0.1775	0.041*	
C36	1.8030 (3)	0.52133 (14)	0.26073 (12)	0.0320 (4)	
H36A	1.8064	0.5115	0.2097	0.048*	
H36B	1.7745	0.4642	0.2902	0.048*	
H36C	1.9192	0.54	0.2727	0.048*	
C37	1.4163 (2)	0.50734 (11)	0.17527 (9)	0.0178 (3)	
C38	1.4923 (2)	0.57287 (12)	0.11282 (9)	0.0224 (4)	
H38A	1.4577	0.5545	0.067	0.034*	
H38B	1.6223	0.5701	0.1135	0.034*	
H38C	1.4455	0.6358	0.1181	0.034*	
C39	1.5068 (3)	0.41108 (12)	0.16890 (10)	0.0250 (4)	
H39A	1.5113	0.3978	0.1186	0.037*	
H39B	1.4387	0.365	0.1988	0.037*	
H39C	1.628	0.4093	0.1853	0.037*	
C40	1.2172 (2)	0.49930 (11)	0.16671 (9)	0.0200 (3)	
H40A	1.1576	0.5608	0.162	0.03*	
H40B	1.1657	0.463	0.2091	0.03*	
H40C	1.201	0.469	0.1235	0.03*	
C41	1.0873 (2)	0.79756 (10)	0.16155 (8)	0.0152 (3)	
O1	1.00361 (16)	0.85776 (8)	0.12106 (6)	0.0185 (2)	
O2	1.06629 (15)	0.80314 (7)	0.23169 (6)	0.0145 (2)	
O3	1.18881 (16)	0.73122 (8)	0.14164 (6)	0.0193 (2)	
Cl1	1.21745 (7)	0.30269 (3)	0.02618 (3)	0.03388 (11)	
Cl2	0.86686 (7)	0.39826 (4)	0.03411 (3)	0.03506 (11)	
C42	1.0296 (3)	0.34275 (17)	−0.02114 (10)	0.0352 (5)	
H42A	0.9774	0.2903	−0.0399	0.042*	
H42B	1.0661	0.3863	−0.0628	0.042*	

### Atomic displacement parameters (Å^2^)

**Table:**

	*U*^11^	*U*^22^	*U*^33^	*U*^12^	*U*^13^	*U*^23^
Pd1	0.01331 (6)	0.00987 (5)	0.01393 (5)	0.00196 (4)	−0.00154 (4)	−0.00259 (4)
Pd2	0.01371 (6)	0.01011 (5)	0.01427 (6)	0.00187 (4)	0.00022 (4)	−0.00223 (4)
P1	0.01167 (19)	0.01141 (17)	0.01529 (18)	0.00150 (14)	−0.00286 (15)	−0.00274 (14)
P2	0.0119 (2)	0.01140 (17)	0.01979 (19)	0.00081 (15)	0.00034 (15)	−0.00310 (14)
C1	0.0232 (9)	0.0167 (7)	0.0157 (7)	0.0048 (6)	−0.0005 (6)	−0.0015 (6)
C2	0.0432 (12)	0.0189 (8)	0.0268 (9)	0.0039 (8)	0.0068 (8)	−0.0001 (7)
C3	0.0669 (17)	0.0276 (10)	0.0266 (10)	0.0072 (10)	0.0140 (10)	0.0092 (8)
C4	0.0765 (19)	0.0384 (12)	0.0172 (9)	0.0134 (12)	0.0076 (10)	−0.0001 (8)
C5	0.0571 (15)	0.0283 (10)	0.0174 (8)	0.0074 (9)	−0.0028 (9)	−0.0077 (7)
C6	0.0256 (9)	0.0194 (8)	0.0149 (7)	0.0048 (7)	−0.0035 (7)	−0.0028 (6)
C7	0.0181 (8)	0.0162 (7)	0.0184 (7)	0.0031 (6)	−0.0028 (6)	−0.0060 (6)
C8	0.0245 (9)	0.0239 (8)	0.0255 (9)	0.0045 (7)	−0.0097 (7)	−0.0125 (7)
C9	0.0237 (10)	0.0200 (8)	0.0403 (11)	−0.0013 (7)	−0.0062 (8)	−0.0158 (7)
C10	0.0218 (9)	0.0136 (7)	0.0363 (10)	−0.0015 (7)	−0.0018 (8)	−0.0054 (7)
C11	0.0164 (8)	0.0139 (7)	0.0233 (8)	0.0029 (6)	−0.0025 (6)	−0.0025 (6)
C12	0.0127 (8)	0.0128 (7)	0.0190 (7)	0.0016 (6)	−0.0008 (6)	−0.0056 (6)
C13	0.0123 (8)	0.0199 (8)	0.0256 (8)	0.0001 (6)	−0.0001 (6)	−0.0023 (6)
C14A	0.0153 (13)	0.0355 (18)	0.0268 (13)	0.0045 (14)	0.0042 (10)	−0.0028 (13)
C15A	0.0212 (15)	0.0266 (14)	0.0462 (19)	−0.0098 (12)	0.0047 (13)	−0.0055 (13)
C16A	0.0167 (13)	0.0341 (16)	0.0327 (15)	0.0069 (11)	−0.0026 (11)	0.0001 (12)
C14B	0.022 (4)	0.036 (5)	0.033 (4)	0.005 (4)	0.007 (3)	0.004 (4)
C15B	0.015 (4)	0.038 (5)	0.048 (5)	−0.011 (3)	0.007 (3)	−0.011 (4)
C16B	0.020 (4)	0.024 (3)	0.045 (5)	0.008 (3)	0.011 (3)	−0.001 (3)
C17	0.0154 (8)	0.0193 (7)	0.0154 (7)	0.0034 (6)	−0.0042 (6)	−0.0021 (6)
C18	0.0180 (8)	0.0210 (8)	0.0152 (7)	0.0006 (6)	−0.0007 (6)	−0.0015 (6)
C19	0.0289 (10)	0.0305 (9)	0.0210 (8)	0.0111 (8)	−0.0082 (7)	−0.0002 (7)
C20	0.0209 (9)	0.0293 (9)	0.0195 (8)	0.0010 (7)	−0.0077 (7)	−0.0085 (7)
C21	0.0179 (8)	0.0228 (8)	0.0162 (7)	0.0046 (7)	0.0000 (6)	−0.0046 (6)
C22	0.0445 (13)	0.0269 (9)	0.0219 (9)	0.0105 (9)	−0.0069 (8)	−0.0072 (7)
C23	0.0649 (17)	0.0401 (12)	0.0258 (10)	0.0203 (11)	−0.0105 (10)	−0.0171 (9)
C24	0.0516 (15)	0.0544 (14)	0.0189 (9)	0.0192 (11)	−0.0043 (9)	−0.0116 (9)
C25	0.0344 (12)	0.0438 (12)	0.0191 (9)	0.0127 (9)	−0.0001 (8)	0.0024 (8)
C26	0.0175 (9)	0.0265 (9)	0.0198 (8)	0.0045 (7)	0.0023 (7)	0.0000 (7)
C27	0.0176 (9)	0.0236 (8)	0.0231 (8)	0.0006 (7)	−0.0025 (7)	0.0053 (7)
C28	0.0289 (11)	0.0357 (11)	0.0322 (10)	0.0004 (9)	0.0016 (8)	0.0137 (8)
C29	0.0402 (13)	0.0320 (11)	0.0428 (12)	−0.0078 (9)	−0.0076 (10)	0.0189 (9)
C30	0.0414 (13)	0.0164 (8)	0.0451 (12)	−0.0019 (8)	−0.0168 (10)	0.0080 (8)
C31	0.0283 (10)	0.0178 (8)	0.0317 (10)	0.0004 (7)	−0.0084 (8)	−0.0008 (7)
C32	0.0157 (8)	0.0158 (7)	0.0241 (8)	−0.0007 (6)	−0.0055 (6)	0.0023 (6)
C33	0.0128 (8)	0.0201 (8)	0.0283 (9)	−0.0021 (6)	0.0004 (7)	−0.0070 (7)
C34	0.0183 (9)	0.0240 (8)	0.0301 (9)	−0.0028 (7)	−0.0054 (7)	−0.0051 (7)
C35	0.0255 (10)	0.0287 (9)	0.0291 (9)	−0.0134 (8)	0.0035 (8)	−0.0050 (7)
C36	0.0137 (9)	0.0330 (10)	0.0519 (12)	0.0038 (8)	−0.0027 (8)	−0.0189 (9)
C37	0.0154 (8)	0.0151 (7)	0.0236 (8)	0.0014 (6)	0.0012 (6)	−0.0074 (6)
C38	0.0206 (9)	0.0242 (8)	0.0225 (8)	−0.0003 (7)	0.0044 (7)	−0.0068 (7)
C39	0.0241 (10)	0.0192 (8)	0.0327 (10)	0.0056 (7)	−0.0022 (8)	−0.0117 (7)
C40	0.0175 (9)	0.0181 (8)	0.0255 (8)	−0.0010 (6)	−0.0021 (7)	−0.0075 (6)
C41	0.0162 (8)	0.0131 (7)	0.0165 (7)	−0.0015 (6)	−0.0004 (6)	−0.0030 (5)
O1	0.0233 (6)	0.0163 (5)	0.0156 (5)	0.0047 (5)	−0.0026 (5)	−0.0025 (4)
O2	0.0179 (6)	0.0121 (5)	0.0131 (5)	0.0029 (4)	−0.0002 (4)	−0.0021 (4)
O3	0.0237 (7)	0.0160 (5)	0.0173 (5)	0.0063 (5)	0.0002 (5)	−0.0033 (4)
Cl1	0.0317 (3)	0.0327 (2)	0.0365 (3)	0.0012 (2)	−0.0078 (2)	0.00191 (19)
Cl2	0.0310 (3)	0.0398 (3)	0.0311 (2)	0.0008 (2)	0.0042 (2)	0.0063 (2)
C42	0.0261 (11)	0.0578 (14)	0.0207 (9)	0.0003 (10)	−0.0019 (8)	−0.0003 (9)

### Geometric parameters (Å, º)

**Table:**

Pd1—C1	1.9658 (16)	C17—C19	1.549 (2)
Pd1—O2	2.1616 (11)	C18—H18A	0.98
Pd1—O1	2.2136 (11)	C18—H18B	0.98
Pd1—P1	2.2185 (4)	C18—H18C	0.98
Pd1—C41	2.5650 (15)	C19—H19A	0.98
Pd2—C21	1.9638 (16)	C19—H19B	0.98
Pd2—O2	2.1452 (11)	C19—H19C	0.98
Pd2—O3	2.1946 (11)	C20—H20A	0.98
Pd2—P2	2.2085 (4)	C20—H20B	0.98
Pd2—C41	2.5522 (16)	C20—H20C	0.98
P1—C12	1.8311 (16)	C21—C26	1.396 (2)
P1—C17	1.8912 (16)	C21—C22	1.398 (2)
P1—C13	1.8947 (17)	C22—C23	1.382 (3)
P2—C32	1.8253 (17)	C22—H22	0.95
P2—C33	1.8789 (18)	C23—C24	1.378 (3)
P2—C37	1.8983 (17)	C23—H23	0.95
C1—C2	1.396 (2)	C24—C25	1.372 (3)
C1—C6	1.400 (2)	C24—H24	0.95
C2—C3	1.384 (3)	C25—C26	1.410 (3)
C2—H2	0.95	C25—H25	0.95
C3—C4	1.382 (3)	C26—C27	1.487 (2)
C3—H3	0.95	C27—C28	1.403 (2)
C4—C5	1.383 (3)	C27—C32	1.410 (2)
C4—H4	0.95	C28—C29	1.377 (3)
C5—C6	1.403 (2)	C28—H28	0.95
C5—H5	0.95	C29—C30	1.372 (3)
C6—C7	1.484 (2)	C29—H29	0.95
C7—C8	1.397 (2)	C30—C31	1.390 (3)
C7—C12	1.413 (2)	C30—H30	0.95
C8—C9	1.387 (3)	C31—C32	1.401 (2)
C8—H8	0.95	C31—H31	0.95
C9—C10	1.378 (3)	C33—C36	1.529 (2)
C9—H9	0.95	C33—C35	1.532 (2)
C10—C11	1.387 (2)	C33—C34	1.538 (2)
C10—H10	0.95	C34—H34A	0.98
C11—C12	1.395 (2)	C34—H34B	0.98
C11—H11	0.95	C34—H34C	0.98
C13—C15B	1.502 (7)	C35—H35A	0.98
C13—C16A	1.519 (3)	C35—H35B	0.98
C13—C14A	1.519 (3)	C35—H35C	0.98
C13—C14B	1.542 (7)	C36—H36A	0.98
C13—C15A	1.559 (3)	C36—H36B	0.98
C13—C16B	1.591 (6)	C36—H36C	0.98
C14A—H14A	0.98	C37—C40	1.534 (2)
C14A—H14B	0.98	C37—C38	1.537 (2)
C14A—H14C	0.98	C37—C39	1.543 (2)
C15A—H15A	0.98	C38—H38A	0.98
C15A—H15B	0.98	C38—H38B	0.98
C15A—H15C	0.98	C38—H38C	0.98
C16A—H16A	0.98	C39—H39A	0.98
C16A—H16B	0.98	C39—H39B	0.98
C16A—H16C	0.98	C39—H39C	0.98
C14B—H14D	0.98	C40—H40A	0.98
C14B—H14E	0.98	C40—H40B	0.98
C14B—H14F	0.98	C40—H40C	0.98
C15B—H15D	0.98	C41—O1	1.2645 (19)
C15B—H15E	0.98	C41—O3	1.2735 (18)
C15B—H15F	0.98	C41—O2	1.3226 (18)
C16B—H16D	0.98	Cl1—C42	1.755 (2)
C16B—H16E	0.98	Cl2—C42	1.772 (2)
C16B—H16F	0.98	C42—H42A	0.99
C17—C20	1.538 (2)	C42—H42B	0.99
C17—C18	1.539 (2)		
			
C1—Pd1—O2	101.55 (5)	H18A—C18—H18B	109.5
C1—Pd1—O1	162.10 (6)	C17—C18—H18C	109.5
O2—Pd1—O1	60.56 (4)	H18A—C18—H18C	109.5
C1—Pd1—P1	84.05 (5)	H18B—C18—H18C	109.5
O2—Pd1—P1	173.47 (3)	C17—C19—H19A	109.5
O1—Pd1—P1	113.82 (3)	C17—C19—H19B	109.5
C1—Pd1—C41	132.57 (6)	H19A—C19—H19B	109.5
O2—Pd1—C41	31.03 (4)	C17—C19—H19C	109.5
O1—Pd1—C41	29.53 (4)	H19A—C19—H19C	109.5
P1—Pd1—C41	143.29 (4)	H19B—C19—H19C	109.5
C21—Pd2—O2	99.28 (6)	C17—C20—H20A	109.5
C21—Pd2—O3	160.34 (6)	C17—C20—H20B	109.5
O2—Pd2—O3	61.13 (4)	H20A—C20—H20B	109.5
C21—Pd2—P2	87.98 (5)	C17—C20—H20C	109.5
O2—Pd2—P2	172.20 (3)	H20A—C20—H20C	109.5
O3—Pd2—P2	111.51 (3)	H20B—C20—H20C	109.5
C21—Pd2—C41	130.46 (6)	C26—C21—C22	119.17 (16)
O2—Pd2—C41	31.20 (4)	C26—C21—Pd2	131.14 (13)
O3—Pd2—C41	29.93 (4)	C22—C21—Pd2	109.66 (12)
P2—Pd2—C41	141.39 (4)	C23—C22—C21	121.45 (18)
C12—P1—C17	108.53 (7)	C23—C22—H22	119.3
C12—P1—C13	107.65 (7)	C21—C22—H22	119.3
C17—P1—C13	111.48 (8)	C24—C23—C22	119.7 (2)
C12—P1—Pd1	104.70 (5)	C24—C23—H23	120.1
C17—P1—Pd1	105.77 (5)	C22—C23—H23	120.1
C13—P1—Pd1	118.20 (5)	C25—C24—C23	119.49 (18)
C32—P2—C33	105.51 (8)	C25—C24—H24	120.3
C32—P2—C37	108.66 (8)	C23—C24—H24	120.3
C33—P2—C37	112.96 (8)	C24—C25—C26	122.14 (18)
C32—P2—Pd2	112.67 (6)	C24—C25—H25	118.9
C33—P2—Pd2	111.56 (5)	C26—C25—H25	118.9
C37—P2—Pd2	105.59 (5)	C21—C26—C25	117.94 (17)
C2—C1—C6	119.12 (15)	C21—C26—C27	124.31 (15)
C2—C1—Pd1	114.19 (13)	C25—C26—C27	117.54 (16)
C6—C1—Pd1	126.63 (12)	C28—C27—C32	117.94 (17)
C3—C2—C1	121.38 (18)	C28—C27—C26	118.37 (17)
C3—C2—H2	119.3	C32—C27—C26	123.44 (15)
C1—C2—H2	119.3	C29—C28—C27	121.9 (2)
C4—C3—C2	119.80 (19)	C29—C28—H28	119
C4—C3—H3	120.1	C27—C28—H28	119
C2—C3—H3	120.1	C30—C29—C28	119.93 (18)
C3—C4—C5	119.47 (18)	C30—C29—H29	120
C3—C4—H4	120.3	C28—C29—H29	120
C5—C4—H4	120.3	C29—C30—C31	119.41 (18)
C4—C5—C6	121.59 (19)	C29—C30—H30	120.3
C4—C5—H5	119.2	C31—C30—H30	120.3
C6—C5—H5	119.2	C30—C31—C32	121.49 (18)
C1—C6—C5	118.56 (16)	C30—C31—H31	119.3
C1—C6—C7	122.96 (14)	C32—C31—H31	119.3
C5—C6—C7	118.19 (15)	C31—C32—C27	118.52 (16)
C8—C7—C12	118.55 (15)	C31—C32—P2	121.72 (14)
C8—C7—C6	119.78 (15)	C27—C32—P2	119.75 (12)
C12—C7—C6	121.56 (15)	C36—C33—C35	110.38 (15)
C9—C8—C7	121.81 (17)	C36—C33—C34	107.79 (15)
C9—C8—H8	119.1	C35—C33—C34	108.05 (14)
C7—C8—H8	119.1	C36—C33—P2	112.38 (12)
C10—C9—C8	119.45 (17)	C35—C33—P2	109.60 (13)
C10—C9—H9	120.3	C34—C33—P2	108.52 (12)
C8—C9—H9	120.3	C33—C34—H34A	109.5
C9—C10—C11	119.91 (16)	C33—C34—H34B	109.5
C9—C10—H10	120	H34A—C34—H34B	109.5
C11—C10—H10	120	C33—C34—H34C	109.5
C10—C11—C12	121.51 (16)	H34A—C34—H34C	109.5
C10—C11—H11	119.2	H34B—C34—H34C	109.5
C12—C11—H11	119.2	C33—C35—H35A	109.5
C11—C12—C7	118.75 (15)	C33—C35—H35B	109.5
C11—C12—P1	121.70 (12)	H35A—C35—H35B	109.5
C7—C12—P1	119.31 (12)	C33—C35—H35C	109.5
C15B—C13—C16A	75.0 (4)	H35A—C35—H35C	109.5
C15B—C13—C14A	127.8 (3)	H35B—C35—H35C	109.5
C16A—C13—C14A	108.86 (19)	C33—C36—H36A	109.5
C15B—C13—C14B	109.4 (4)	C33—C36—H36B	109.5
C16A—C13—C14B	126.3 (4)	H36A—C36—H36B	109.5
C16A—C13—C15A	109.0 (2)	C33—C36—H36C	109.5
C14A—C13—C15A	107.6 (2)	H36A—C36—H36C	109.5
C14B—C13—C15A	82.8 (4)	H36B—C36—H36C	109.5
C15B—C13—C16B	108.4 (4)	C40—C37—C38	109.34 (14)
C14A—C13—C16B	80.2 (3)	C40—C37—C39	106.86 (14)
C14B—C13—C16B	104.3 (4)	C38—C37—C39	107.75 (13)
C15A—C13—C16B	138.9 (4)	C40—C37—P2	105.47 (11)
C15B—C13—P1	114.1 (3)	C38—C37—P2	108.00 (11)
C16A—C13—P1	112.88 (14)	C39—C37—P2	119.15 (12)
C14A—C13—P1	111.55 (15)	C37—C38—H38A	109.5
C14B—C13—P1	112.8 (3)	C37—C38—H38B	109.5
C15A—C13—P1	106.77 (14)	H38A—C38—H38B	109.5
C16B—C13—P1	107.2 (3)	C37—C38—H38C	109.5
C13—C14A—H14A	109.5	H38A—C38—H38C	109.5
C13—C14A—H14B	109.5	H38B—C38—H38C	109.5
C13—C14A—H14C	109.5	C37—C39—H39A	109.5
C13—C15A—H15A	109.5	C37—C39—H39B	109.5
C13—C15A—H15B	109.5	H39A—C39—H39B	109.5
C13—C15A—H15C	109.5	C37—C39—H39C	109.5
C13—C16A—H16A	109.5	H39A—C39—H39C	109.5
C13—C16A—H16B	109.5	H39B—C39—H39C	109.5
C13—C16A—H16C	109.5	C37—C40—H40A	109.5
C13—C14B—H14D	109.5	C37—C40—H40B	109.5
C13—C14B—H14E	109.5	H40A—C40—H40B	109.5
H14D—C14B—H14E	109.5	C37—C40—H40C	109.5
C13—C14B—H14F	109.5	H40A—C40—H40C	109.5
H14D—C14B—H14F	109.5	H40B—C40—H40C	109.5
H14E—C14B—H14F	109.5	O1—C41—O3	126.51 (14)
C13—C15B—H15D	109.5	O1—C41—O2	117.03 (13)
C13—C15B—H15E	109.5	O3—C41—O2	116.45 (14)
H15D—C15B—H15E	109.5	O1—C41—Pd2	174.15 (11)
C13—C15B—H15F	109.5	O3—C41—Pd2	59.29 (8)
H15D—C15B—H15F	109.5	O2—C41—Pd2	57.16 (7)
H15E—C15B—H15F	109.5	O1—C41—Pd1	59.65 (8)
C13—C16B—H16D	109.5	O3—C41—Pd1	173.67 (12)
C13—C16B—H16E	109.5	O2—C41—Pd1	57.39 (7)
H16D—C16B—H16E	109.5	Pd2—C41—Pd1	114.53 (6)
C13—C16B—H16F	109.5	C41—O1—Pd1	90.82 (9)
H16D—C16B—H16F	109.5	C41—O2—Pd2	91.64 (9)
H16E—C16B—H16F	109.5	C41—O2—Pd1	91.58 (9)
C20—C17—C18	108.11 (13)	Pd2—O2—Pd1	176.28 (6)
C20—C17—C19	106.79 (14)	C41—O3—Pd2	90.78 (9)
C18—C17—C19	108.83 (14)	Cl1—C42—Cl2	111.76 (11)
C20—C17—P1	106.62 (11)	Cl1—C42—H42A	109.3
C18—C17—P1	106.29 (11)	Cl2—C42—H42A	109.3
C19—C17—P1	119.73 (11)	Cl1—C42—H42B	109.3
C17—C18—H18A	109.5	Cl2—C42—H42B	109.3
C17—C18—H18B	109.5	H42A—C42—H42B	107.9
			
C1—Pd1—P1—C12	57.47 (7)	C41—Pd2—C21—C22	−30.19 (17)
O1—Pd1—P1—C12	−121.64 (6)	C26—C21—C22—C23	1.5 (3)
C41—Pd1—P1—C12	−119.08 (8)	Pd2—C21—C22—C23	179.60 (19)
C1—Pd1—P1—C17	172.03 (8)	C21—C22—C23—C24	0.8 (4)
O1—Pd1—P1—C17	−7.09 (7)	C22—C23—C24—C25	−1.6 (4)
C41—Pd1—P1—C17	−4.52 (9)	C23—C24—C25—C26	0.1 (4)
C1—Pd1—P1—C13	−62.29 (8)	C22—C21—C26—C25	−2.8 (3)
O1—Pd1—P1—C13	118.60 (7)	Pd2—C21—C26—C25	179.48 (15)
C41—Pd1—P1—C13	121.16 (9)	C22—C21—C26—C27	−177.42 (18)
C21—Pd2—P2—C32	40.09 (8)	Pd2—C21—C26—C27	4.9 (3)
O3—Pd2—P2—C32	−142.55 (7)	C24—C25—C26—C21	2.1 (3)
C41—Pd2—P2—C32	−144.76 (8)	C24—C25—C26—C27	177.1 (2)
C21—Pd2—P2—C33	−78.37 (8)	C21—C26—C27—C28	−154.21 (19)
O3—Pd2—P2—C33	98.99 (7)	C25—C26—C27—C28	31.2 (3)
C41—Pd2—P2—C33	96.77 (8)	C21—C26—C27—C32	31.6 (3)
C21—Pd2—P2—C37	158.55 (8)	C25—C26—C27—C32	−142.96 (19)
O3—Pd2—P2—C37	−24.08 (7)	C32—C27—C28—C29	5.3 (3)
C41—Pd2—P2—C37	−26.30 (8)	C26—C27—C28—C29	−169.2 (2)
O2—Pd1—C1—C2	−52.97 (15)	C27—C28—C29—C30	2.5 (3)
O1—Pd1—C1—C2	−52.2 (3)	C28—C29—C30—C31	−5.7 (3)
P1—Pd1—C1—C2	130.44 (15)	C29—C30—C31—C32	1.0 (3)
C41—Pd1—C1—C2	−52.36 (18)	C30—C31—C32—C27	6.7 (3)
O2—Pd1—C1—C6	124.33 (16)	C30—C31—C32—P2	−172.66 (15)
O1—Pd1—C1—C6	125.09 (19)	C28—C27—C32—C31	−9.6 (3)
P1—Pd1—C1—C6	−52.27 (16)	C26—C27—C32—C31	164.54 (17)
C41—Pd1—C1—C6	124.93 (15)	C28—C27—C32—P2	169.78 (15)
C6—C1—C2—C3	0.6 (3)	C26—C27—C32—P2	−16.0 (2)
Pd1—C1—C2—C3	178.08 (18)	C33—P2—C32—C31	−83.13 (16)
C1—C2—C3—C4	−2.3 (4)	C37—P2—C32—C31	38.27 (17)
C2—C3—C4—C5	1.4 (4)	Pd2—P2—C32—C31	154.91 (14)
C3—C4—C5—C6	1.3 (4)	C33—P2—C32—C27	97.47 (15)
C2—C1—C6—C5	2.0 (3)	C37—P2—C32—C27	−141.13 (14)
Pd1—C1—C6—C5	−175.16 (15)	Pd2—P2—C32—C27	−24.48 (16)
C2—C1—C6—C7	−171.70 (18)	C32—P2—C33—C36	73.16 (15)
Pd1—C1—C6—C7	11.1 (3)	C37—P2—C33—C36	−45.41 (16)
C4—C5—C6—C1	−3.0 (3)	Pd2—P2—C33—C36	−164.17 (12)
C4—C5—C6—C7	171.1 (2)	C32—P2—C33—C35	−163.72 (12)
C1—C6—C7—C8	−150.28 (18)	C37—P2—C33—C35	77.71 (13)
C5—C6—C7—C8	36.0 (3)	Pd2—P2—C33—C35	−41.06 (13)
C1—C6—C7—C12	33.6 (3)	C32—P2—C33—C34	−45.94 (13)
C5—C6—C7—C12	−140.15 (18)	C37—P2—C33—C34	−164.51 (11)
C12—C7—C8—C9	0.5 (3)	Pd2—P2—C33—C34	76.73 (12)
C6—C7—C8—C9	−175.77 (16)	C32—P2—C37—C40	72.50 (12)
C7—C8—C9—C10	0.7 (3)	C33—P2—C37—C40	−170.78 (11)
C8—C9—C10—C11	−0.6 (3)	Pd2—P2—C37—C40	−48.60 (11)
C9—C10—C11—C12	−0.6 (3)	C32—P2—C37—C38	−170.68 (11)
C10—C11—C12—C7	1.7 (2)	C33—P2—C37—C38	−53.96 (13)
C10—C11—C12—P1	−172.58 (13)	Pd2—P2—C37—C38	68.22 (12)
C8—C7—C12—C11	−1.7 (2)	C32—P2—C37—C39	−47.43 (15)
C6—C7—C12—C11	174.52 (15)	C33—P2—C37—C39	69.29 (15)
C8—C7—C12—P1	172.80 (13)	Pd2—P2—C37—C39	−168.53 (12)
C6—C7—C12—P1	−11.0 (2)	C21—Pd2—C41—O3	177.75 (10)
C17—P1—C12—C11	21.43 (15)	O2—Pd2—C41—O3	179.84 (15)
C13—P1—C12—C11	−99.37 (14)	P2—Pd2—C41—O3	4.13 (13)
Pd1—P1—C12—C11	134.03 (12)	C21—Pd2—C41—O2	−2.09 (13)
C17—P1—C12—C7	−152.87 (13)	O3—Pd2—C41—O2	−179.84 (15)
C13—P1—C12—C7	86.33 (14)	P2—Pd2—C41—O2	−175.71 (6)
Pd1—P1—C12—C7	−40.27 (13)	C21—Pd2—C41—Pd1	−3.81 (11)
C12—P1—C13—C15B	169.7 (4)	O2—Pd2—C41—Pd1	−1.73 (6)
C17—P1—C13—C15B	50.7 (4)	O3—Pd2—C41—Pd1	178.44 (14)
Pd1—P1—C13—C15B	−72.1 (4)	P2—Pd2—C41—Pd1	−177.43 (2)
C12—P1—C13—C16A	86.48 (18)	C1—Pd1—C41—O1	179.90 (10)
C17—P1—C13—C16A	−32.44 (19)	O2—Pd1—C41—O1	−178.96 (15)
Pd1—P1—C13—C16A	−155.30 (16)	P1—Pd1—C41—O1	−4.76 (13)
C12—P1—C13—C14A	−36.5 (2)	C1—Pd1—C41—O2	−1.14 (13)
C17—P1—C13—C14A	−155.39 (18)	O1—Pd1—C41—O2	178.96 (15)
Pd1—P1—C13—C14A	81.75 (19)	P1—Pd1—C41—O2	174.19 (6)
C12—P1—C13—C14B	−64.6 (4)	C1—Pd1—C41—Pd2	0.58 (11)
C17—P1—C13—C14B	176.4 (4)	O2—Pd1—C41—Pd2	1.72 (6)
Pd1—P1—C13—C14B	53.6 (4)	O1—Pd1—C41—Pd2	−179.32 (14)
C12—P1—C13—C15A	−153.80 (17)	P1—Pd1—C41—Pd2	175.92 (2)
C17—P1—C13—C15A	87.28 (18)	O3—C41—O1—Pd1	178.24 (16)
Pd1—P1—C13—C15A	−35.58 (18)	O2—C41—O1—Pd1	−0.99 (14)
C12—P1—C13—C16B	49.6 (4)	C1—Pd1—O1—C41	−0.2 (2)
C17—P1—C13—C16B	−69.3 (4)	O2—Pd1—O1—C41	0.62 (9)
Pd1—P1—C13—C16B	167.8 (4)	P1—Pd1—O1—C41	176.89 (9)
C12—P1—C17—C20	169.61 (11)	O1—C41—O2—Pd2	179.15 (13)
C13—P1—C17—C20	−71.99 (12)	O3—C41—O2—Pd2	−0.16 (15)
Pd1—P1—C17—C20	57.72 (11)	Pd1—C41—O2—Pd2	178.14 (6)
C12—P1—C17—C18	54.44 (12)	O1—C41—O2—Pd1	1.01 (15)
C13—P1—C17—C18	172.84 (10)	O3—C41—O2—Pd1	−178.29 (13)
Pd1—P1—C17—C18	−57.45 (11)	Pd2—C41—O2—Pd1	−178.14 (6)
C12—P1—C17—C19	−69.22 (15)	C21—Pd2—O2—C41	178.39 (10)
C13—P1—C17—C19	49.18 (16)	O3—Pd2—O2—C41	0.09 (9)
Pd1—P1—C17—C19	178.90 (13)	C1—Pd1—O2—C41	179.14 (10)
O2—Pd2—C21—C26	146.55 (17)	O1—Pd1—O2—C41	−0.59 (9)
O3—Pd2—C21—C26	150.98 (15)	O1—C41—O3—Pd2	−179.07 (16)
P2—Pd2—C21—C26	−36.33 (17)	O2—C41—O3—Pd2	0.15 (14)
C41—Pd2—C21—C26	147.65 (15)	C21—Pd2—O3—C41	−5.1 (2)
O2—Pd2—C21—C22	−31.29 (15)	O2—Pd2—O3—C41	−0.10 (9)
O3—Pd2—C21—C22	−26.9 (3)	P2—Pd2—O3—C41	−177.23 (9)
P2—Pd2—C21—C22	145.83 (14)		

### Hydrogen-bond geometry (Å, º)

**Table:**

*D*—H···*A*	*D*—H	H···*A*	*D*···*A*	*D*—H···*A*
C18—H18*A*···O1	0.98	2.53	3.362 (2)	143
C20—H20*C*···O1	0.98	2.27	3.182 (2)	154
C22—H22···O2	0.95	2.43	3.030 (2)	121
C35—H35*A*···O2^i^	0.98	2.43	3.358 (2)	159
C38—H38*C*···O3	0.98	2.37	3.223 (2)	146
C40—H40*A*···O3	0.98	2.51	3.377 (2)	147
C42—H42*A*···O3^ii^	0.99	2.40	3.190 (2)	136

Symmetry codes: (i) *x*+1, *y*, *z*; (ii) −*x*+2, −*y*+1, −*z*.
